# Conductometric Soot Sensor for Automotive Exhausts: Initial Studies

**DOI:** 10.3390/s100301589

**Published:** 2010-03-01

**Authors:** Gunter Hagen, Constanze Feistkorn, Sven Wiegärtner, Andreas Heinrich, Dieter Brüggemann, Ralf Moos

**Affiliations:** 1 Functional Materials, Bayreuth Engine Research Center (BERC), University of Bayreuth, 95440 Bayreuth, Germany; E-Mail: Functional.Materials@Uni-Bayreuth.de (R.M.); 2 Engineering Thermodynamics and Transport Processes, Bayreuth Engine Research Center (BERC), University of Bayreuth, 95440 Bayreuth, Germany; E-Mail: LTTT@Uni-Bayreuth.de (D.B.)

**Keywords:** on-board diagnosis (OBD), diesel particulate filter (DPF), emission legislation, diesel particulate matter (PM)

## Abstract

In order to reduce the tailpipe particulate matter emissions of Diesel engines, Diesel particulate filters (DPFs) are commonly used. Initial studies using a conductometric soot sensor to monitor their filtering efficiency, *i.e.*, to detect a malfunction of the DPF, are presented. The sensors consist of a planar substrate equipped with electrodes on one side and with a heater on the other. It is shown that at constant speed-load points, the time until soot percolation occurs or the resistance itself are reproducible means that can be well correlated with the filtering efficiency of a DPF. It is suggested to use such a sensor setup for the detection of a DPF malfunction.

## 1. Introduction

Due to increasing fuel costs and concerns about global warming, the automotive industry is constantly improving the combustion techniques of their engines. In particular novel Diesel engines are highly effective, but their NO_x_ and Diesel particulate emissions exceed by far the legal limits without appropriate exhaust gas aftertreatment systems. Particulate matter (PM) from Diesel engines consists of aggregated carbon particles (soot) with adhered organic compounds like hydrocarbons, and smaller amounts of inorganic components like sulfates or ashes. Diesel particulates may affect human health due to their size in the range of some tens of nm, meaning that they are alveolar; therefore, Diesel PM emissions are limited with steadily tightening emission limits [1].

Diesel particulate filters (DPFs) are the commonly used exhaust gas aftertreatment devices that reduce the tailpipe PM emissions by mass as well as by particle number [2]. Typically, DPFs are porous ceramic wall-flow filters with alternate plugged channels. The exhaust is forced through the very porous channel walls, wherein the particulates are trapped. A more detailed overview on DPF technology and markets can be found in [1–4]. With increasing soot load, the flow resistance increases, too. To avoid clogging and increasing fuel consumption, the particulate filter must be regenerated at certain intervals, typically when a soot load of several grams per liter catalyst volume is deposited. During a typical soot regeneration phase, the carbonaceous soot is oxidized. Using exhaust gas oxygen as an oxidation means, the DPF has to be heated to about 600 °C for a secure soot removal. By adding an oxidation catalyst in front of the DPF and/or by catalytically coating the DPF, NO_2_ is formed from NO_x_. Since NO_2_ is much more reactive than O_2_, a DPF temperature of only about 200 °C is required in that case [5].

In serial applications, a differential pressure sensor that determines the pressure difference up- and downstream of the DPF is installed in the exhaust pipe. Applying the pressure difference signal to a pressure model at specific volumetric flow rates, a determination can be made when the filter requires soot regeneration and to some extent also whether it is defect or ash-clogged [6]. Other methods to determine the soot loading are measurements of the electrical impedance of the DPF itself during operation [7], a method that has also been applied to measure the NO_x_ loading and the degree of sulfurization of lean NO_x_ traps [8], to determine the amount of stored oxygen in three-way catalysts [9], and to obtain information on the ammonia loading of zeolite SCR catalysts [10]. It has also been shown that coke deposits in industrial fixed bed catalysts can be directly and in-situ monitored by impedance spectroscopy of a representative catalyst pellet [11]. Recently, initial results suggest measuring the soot loading of DPFs even wireless with a radio frequency-based system [12].

Like all other exhaust gas aftertreatment systems, the efficiency of the DPF has to be monitored (On-Board-Diagnosis, OBD). For three way catalysts, which are applied in stoichiometrically operated gasoline engines, additional lambda probes are installed downstream of the catalyst for that purpose [4,13,14]. The efficiency of NO_x_ storage catalysts can be monitored with NO_x_ sensors, whereas for urea SCR systems, wideband lambda probes, NO_x_ sensors, or ammonia sensors are taken into consideration [15,16]. Similar to the classical catalyst devices, it is clear that a malfunction of a DPF has to be indicated to the driver. For that reason, several types of soot sensors are in discussion [6]. Despite its importance, only very few information is available in the open literature concerning that topic. Recently, Riegel and Klett have reviewed some suggested principles [17]. Besides the conductometric principle, in which the PM amount is calculated from the conductivity of the particles that are deposited between an interdigital electrode structure, optical sensors, and discharge sensors are discussed. Contamination may be a drawback of the optical principle, whereas the high voltage may be prohibitively costly for the discharge principle. Based on the current knowledge, Ref. [17] considered the conductometric principle as the best choice for a serial application of an OBD-PM sensor for DPF diagnosis.

However, it should be noted here that in the open literature results of such a sensor are neither published nor was it investigated whether such a sensor can really detect a DPF malfunction. The aim of this paper is to provide first results obtained in a dynamometer test bench to open the scientific discussion on that topic. Especially, it shall be investigated whether it is in principle suitable to detect a decreasing soot filtering efficiency with such a sensor setup.

## 2. Experimental

**Figure 1.**
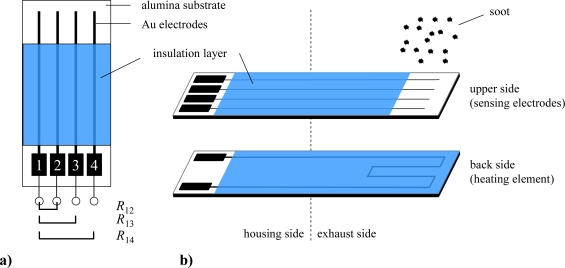
Sketch of the setup of the planar soot sensor. (a) Top view and meaning of the measured resistances *R*_12_, *R*_13_, *R*_14_. (b) Details of the sensor setup with respect to top side and back side.

The sensor setup to investigate the effect of soot particles between electrodes is illustrated in Figure 1. On one side of a planar alumina ceramic substrate (CeramTec Rubalit 708S, 96% Al_2_O_3_; 6.35 mm × 50.8 mm × 635 μm), a platinum resistance structure serving as a heater was screen-printed and fired. The heater and the contact tracks were covered by an electrically insulating glass ceramic film (DuPont, QM42) to protect the tracks from being shortened by soot deposition and to avoid soot burning directly at the platinum heater. On the other side of the substrate, four gold electrodes (each 500 μm broad and with 1.5 mm spacing) were applied and also covered by a glass ceramic film. A defined area (5 mm × 5.5 mm) was left uncovered for soot loading. The sensor was installed in a suitable housing without a protective cap and is mounted into the exhaust pipe using an 18 mm boss for lambda probes. Care was taken to face the electrode side exactly perpendicular to the exhaust flow in each measurement.

**Figure 2.**
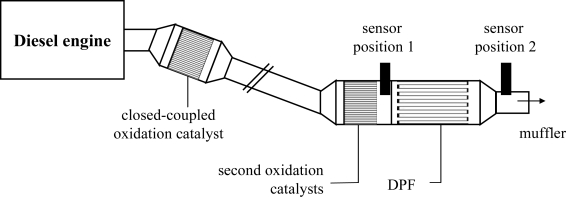
Setup of engine and sensor positions. The second test with a sensor at “sensor position 2” was conducted without the second oxidation catalyst.

All tests were carried out in an engine test bench (dynamometer) on an Audi V6, 3.0 l TDI engine. It is equipped with a close-coupled oxidation catalyst for hydrocarbon conversion during cold start. The purpose of the second oxidation catalyst is to oxidize NO to NO_2_ to support soot regeneration. An uncoated silicon carbide ceramic DPF (300 cpsi) filters the particulate matter. During all tests, the engine was operated at 1,000 rpm with a 40% load (approx. 300 nm). It had been known from previous investigations that at this speed-load-point the engine produces enough soot in a reliable amount but the exhaust temperature is that low that no soot oxidation on the planar sensor substrate occurs. For the first tests, the sensors were screwed into the tailpipe at “sensor position 1” directly between the second oxidation catalyst and the DPF as shown in Figure 2. The four top contacts of the sensors were connected to a Keithley 2,700 Multichannel-Digital-Multimeter (DMM), and the resistances between the electrodes, *R*_12_, *R*_13_, and *R*_14_, as depicted in Figure 1, were measured in the Auto Range mode. After a certain period of soot loading, we heated the sensors up to 600 °C to burn off the deposited soot (heater resistance 4 wire-mode ∼4 Ω at room temperature, *U* = 7 V, *I* = 0.65 A). The sensors got regenerated and were ready for a subsequent soot loading.

**Figure 3.**
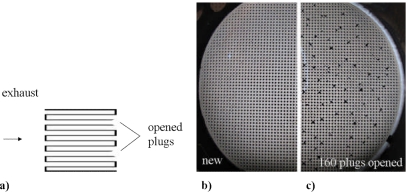
Diesel particulate filter with opened channels. (a) Principle. (b) Photography of the original device with closed channels. (c) Photography of the device with 160 opened plugs.

Since it shall be investigated whether in principle a decreasing soot filtering efficiency can be detected, sensors were also installed downstream of the DPF at “sensor position 2”. The decreasing filtering efficiency was simulated by subsequently opening some of the plugged channels. For these tests, we used a special setup without the second oxidation catalyst, since mounting and dismounting of the DPF was easier. The principle is depicted in Figure 3a.

Due to the opening of the plugs, the exhaust flows through the channels without forcing the exhaust through the porous ceramic walls. Therefore, the filtration efficiency decreases. Figures 3b,c are photographs of the original DPF and of the opened device, respectively.

## 3. Results and Discussion

### 3.1. Sensor at position 1 (results)

**Figure 4.**
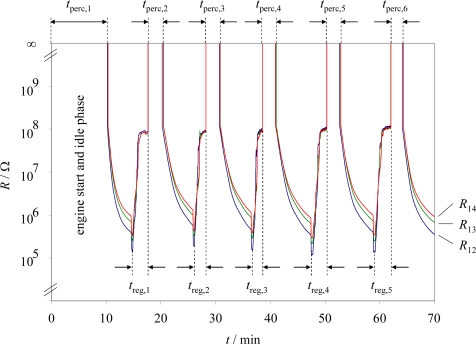
Resistances *R*_12_, *R*_13_, and *R*_14_ during periodic loading and regeneration. Tests were conducted at sensor position 1.

The results of the first experimental series with the conductometric soot sensor located at sensor position 1 are shown in Figure 4. The sensor resistances *R*_12_, *R*_13_, and *R*_14_ are plotted logarithmically *vs.* time. Right before engine start, the sensor was burned free with the internal heater. When the sensor was cooled down, the engine was started (*t* = 0). At this point, the sensor resistances could not have been measured (overflow of the DMM). At first, after starting the engine, we had a 500 s period of idle running to check whether the sensor and the data logging operate correctly. After that, the load was increased to the above-mentioned constant operation point of 300 nm.

As soon as the engine started, the sensor got soot loaded, and after the time span *t*_perc,1_ at about 10 min, the resistance became measurable. The time span *t*_perc,1_ included the engine start and an idle running phase with a low soot formation. After *R*_14_ reached 1 MΩ (approximately) for the first time, the sensor was heated and the soot burned off. During this first regeneration period, *t*_reg,1_, the resistance decreased initially but then increased to a value of about 100 MΩ. After the sensor had reached a constant resistance value, the heater was switched off, the sensor cooled down to exhaust temperature, and soot deposition started again. At this point, the resistances could not be measured since there is no soot on the sensor. After a distinct percolation time interval, *t*_perc,2_, the sensor resistances became measurable again. They constantly decreased until the next regeneration was initiated, when *R*_14_ reached 1 MΩ. Again, after reaching a constant resistance value, the heating power was turned off, and the sensor cooled down to exhaust temperature. This is the beginning of the next soot-collecting phase, and so on. These test cycles were repeated five times. The sensor temperature during soot loading at this position during the first experiment (this is also the gas temperature) was about 290 °C.

### 3.2. Sensor at position 1 (discussion)

**Figure 5.**
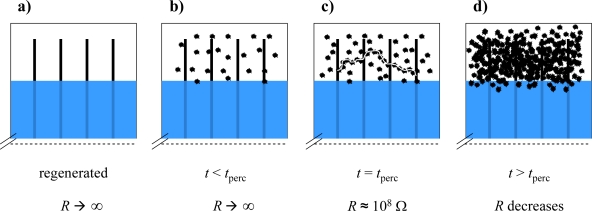
Schematic explanation of the resistance behavior during loading. For the meaning of (a) to (d) see text.

The results of the test series at sensor position 1 can be explained as follows. At the beginning of the second percolation time interval, *t*_perc,2_ at about 18 min, the sensor is free of soot deposit and since the sensor is not heated, its temperature is approximately exhaust temperature (290 °C). At that temperature, the alumina substrate can be considered as a very good electrical insulator, with a resistivity above 10^11^ Ωcm [18]. Therefore, the sensor resistances are too high ohmic to be measured with the used conventional DMM (Figure 5a). Clearly, the sensor starts to collect soot, as depicted in Figure 5. At the beginning, only scattered soot particles are deposited (Figure 5b). With increasing time, the first conduction paths form, and the sensor reaches the percolation threshold (Figure 5c). That is when the resistances decrease suddenly by decades. If one magnifies Figure 4, one can even notice that *R*_12_ reaches the percolation threshold mostly faster than *R*_13_ and *R*_14_. This agrees with the assumption of formed paths that initiate the sudden resistance decrease. If the initial path forms first between electrode 1 and 2, *R*_12_ should be the first sensor where the percolation occurs. If, however, the conductive path forms first between electrode 2 and 3 or 3 and 4, due to the serial connection of *R*_12_, *R*_23_, and *R*_34_, the percolation time of *R*_13_ or *R*_14_ can never be lower than that of *R*_12_.

From Figure 5d it becomes obvious that the more soot gets deposited on the sensor, the more the resistances decrease, mainly since more conductive paths form and the soot loading becomes more dense, but also because the soot film thickness increases. The observed behavior *R*_12_ < *R*_13_ < *R*_14_ can be expected and is attributed to a simple effect of the electrode distances.

It should be noted that the definition of the percolation time is not exact. According to the generalized effective media theory [19,20], the sensor resistance does not decrease jump-like when reaching the percolation threshold but a small increase of the conductive particle fraction (here in an electrically insulating exhaust gas “matrix”) reduces the total resistivity by decades. Hence, to arbitrarily define the end of the percolation when the resistance *R*_12_ falls below the overflow reading of the DMM is rather convenient than exact. Nevertheless, it has to be emphasized that this definition leads to a reproducible measurand average from *t*_perc,2_ to *t*_perc,6_ of 142.3 s with a standard deviation of 12.4 s.

After turning on the heater power, the resistance decreases for a few seconds. This is due to the increased conductivity of the soot with increasing temperature, an effect that has already been observed in literature when the coke formation in fixed bed catalysts has been in-situ determined [21]. Investigations on fullerene soot also depict increasing conductivity at higher temperatures [22]. As soon as the soot ignition temperature is reached, the thin soot film is oxidized and the resistances increase rapidly. At a first glance, one might have expected that the resistance of the soot-free heated device is almost undetectable high, *i.e.*, that the utilized conventional DMM shows an “overflow” value. However, at the soot burn-off temperature of 600 °C, the resistivity of the 96% alumina substrate decreases to about ρ ≈ 10^8^ Ωcm [18]. A rough estimation using the geometry of the samples leads to a resistance in the order of 4 × 10^8^ Ω, which is in good agreement with the measured values. Hence, the plateau region right before turning off the heater power is not a result of the soot, but is due to the non-neglecting conductivity of the utilized alumina substrate. As a conclusion of this section, it turned out that the percolation time of a loading cycle might be an indicator for the detection of the filtering efficiency.

### 3.3. Sensor at position 2 (results and discussion)

**Figure 6.**
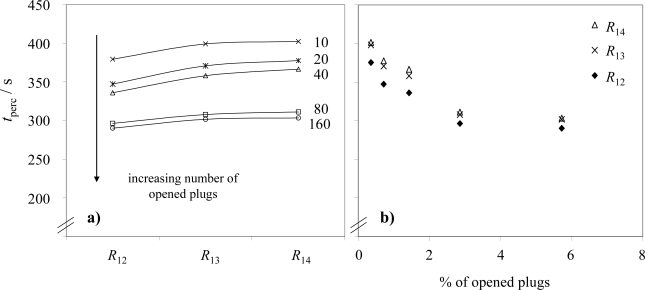
Percolation times of a planar soot sensor downstream of the DPF (a) in dependence of the electrode structure and (b) in dependence of the number of opened plugs.

In the subsequent tests, sensors were installed downstream of the DPF at sensor position 2 to investigate whether a decaying DPF filtration efficiency can be detected. The initial tests were performed with a malfunction-free DPF, *i.e.*, the channels remained plugged. Since in this case almost no soot is in the exhaust downstream of the DPF, the resistances remained in the overflow region. However, after opening 10 channels of the DPF, it was possible to determine the percolation times. As shown in Figure 6a, the first percolation interval, *t*_perc,1_ decreased with an increasing number of opened plugs. During all these experiments, the constant operation point (300 Nm) was attained about 30 s after starting the engine without the idle phases as described in the initial test in Sec. 3.1. We evaluated the first percolation interval in order to reduce the length of time for the entire tests. In agreement with section 3.2, the percolation time for the resistance *R*_12_, has always remained lower or equal than for *R*_13_ or *R*_14_, and the percolation time for the resistance *R*_13_ has always been lower or equal than for *R*_14_. Since the number of opened plugs can be seen as an indication for the amount of non-filtered exhaust, one might expect a strong decrease of *t*_perc_ for a low number of openings. With an increasing number of opened plugs not only the filtered area is reduced, but due to the much lower backpressure in the opened channels, it is assumed that also a high proportion of the exhaust flows through these channels. As a result, the particle increase is disproportionately high when only a few plugs are opened. This behavior is reflected in Figure 6b.

## 4. Conclusions and Outlook

In the present assessment study, we present initial results on soot detection in the automotive exhaust. In fact, the used setup is very simple. The resistance between screen-printed Au electrodes is a measure for deposited soot on top of the device. One may either use the time until a percolation of soot particles occurs between the electrodes (the resistance steps down to measurable values when the percolation threshold is reached) or the resistance itself (which depends on the amount of soot or on the thickness of the deposited soot film respectively) to correlate with the status of soot loading of a DPF or to check whether the DPF has a malfunction.

It is evident that many points are unsettled. The signal (e.g., *t*_perc_) has to be correlated with the actual soot loading of the DPF. The soot has to be characterized in detail (morphology, particle size, particle number, ashes, its degree of moisture), also at different speed and load levels. The effect of the sensors mounting orientation (directly faced or parallel to the exhaust flow), the sensor temperature (depositing kinetics) or measuring effects (electric fields due to high voltage in the region prior to percolation) have to be investigated. Of course, it has to be investigated whether all parts of the sensor device are long-term stable in the automotive exhaust. Especially one should keep an eye on the material for the electrodes, since the high temperatures that occur during soot regeneration might affect them.
